# Marfan Syndrome: A Case Report

**DOI:** 10.1155/2012/595343

**Published:** 2012-12-04

**Authors:** Rajendran Ganesh, Rajendran Vijayakumar, Haridoss Selvakumar

**Affiliations:** Department of Pedodontics, SRM Dental College, SRM University, Chennai 600078, India

## Abstract

Marfan syndrome is an autosomal dominant systemic disorder of the connective tissue. Children affected by the Marfan syndrome carry a mutation in one of their two copies of the gene that encodes the connective tissue protein fibrillin-1. Marfan syndrome affects most organs and tissues, especially the skeleton, lungs, eyes, heart, and the large blood vessel that distributes blood from the heart to the rest of the body. A case report of Marfan syndrome has been reported with oral features. The dental problems of the child were treated under general anesthesia and a one-month review showed intact stainless steel crowns' restorations and no signs of secondary caries.

## 1. Introduction

Marfan syndrome is an autosomal dominant systemic disorder of connective tissue [1]. Children affected by the Marfan syndrome carry a mutation in one of their two copies of the gene that encodes the connective tissue protein fibrillin-1 (FBN 1) [2].

It mostly affects skeleton, lungs, eyes, heart, and the aorta [3]. Affected individuals often are tall and slender, have arachnodactyly, scoliosis, and either a pectus excavatum, pectus carinatum, or ectopia lentis in eyes [4]. The incidence of mitral prolapse in such patients is essentially equal in children and adults of the same sex [5].

Worldwide, the incidence of Marfan syndrome is approximately 7–17/100,000 [6]. The incidence of aortic dilatation and mitral prolapse in patients with Marfan's syndrome was essentially equal in children and adults of the same sex. The oral cavity shows high-arched palate that result from a narrow maxilla [7]. Connective tissue disorders have been associated with severe periodontitis [8]. Here I am presenting a case report of Marfan syndrome with dental decay which is successfully treated.

## 2. Case Report

A 6-year-old girl reported along with her mother to the Private Dental Clinic, Chennai, with a chief complaint of decayed teeth in both upper and lower front and back teeth region. The medical history revealed that she has Marfan syndrome with tricuspid and mitral valve prolapse and dilation of aorta.

**Figure 1:**
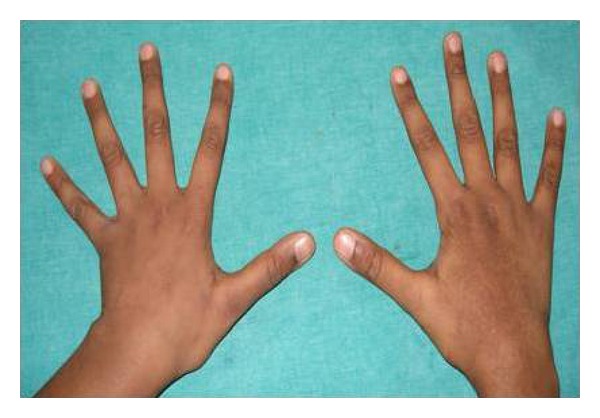
Photographs showing arachnodactyly.

**Figure 2:**
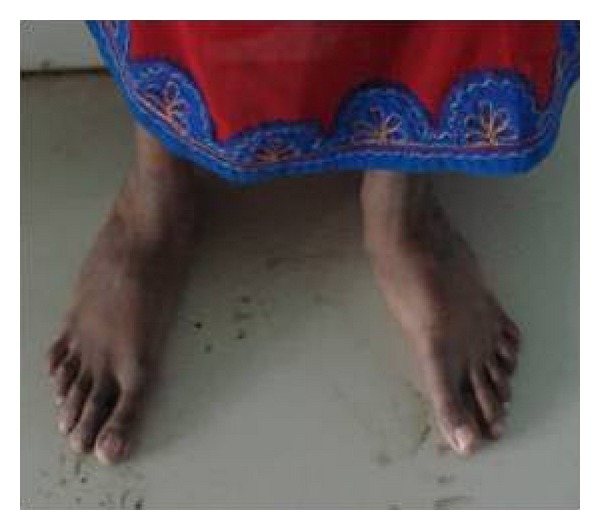
Photographs showing arachnodactyly.

**Figure 3:**
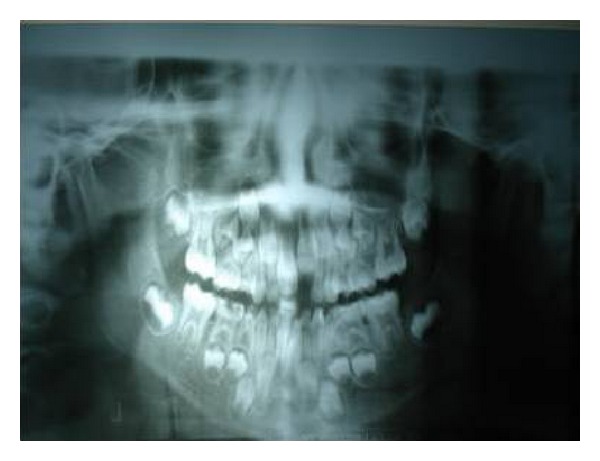
OPG showing grossly decayed 54 and 61, 83, 84, and 85 showed dental caries involving the pulp in 54, 74.

**Figure 4:**
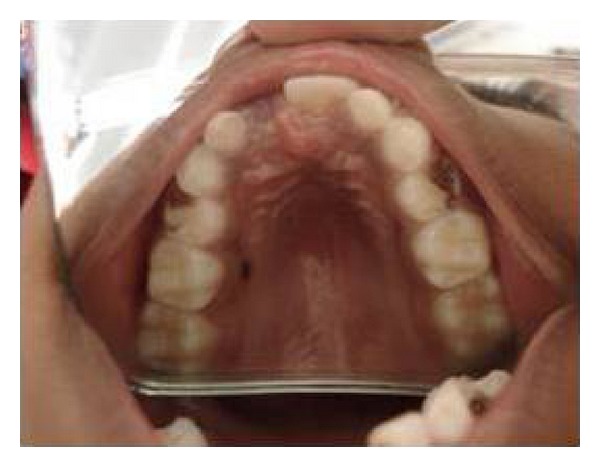
Intraoral preoperative photographs showing dental caries in 54 and 64.

**Figure 5:**
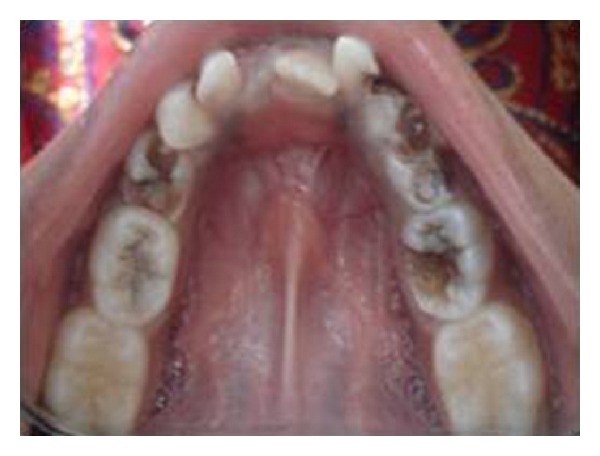
Intraoral preoperative photograph showing decayed 74, 83, 84, and 85.

**Figure 6:**
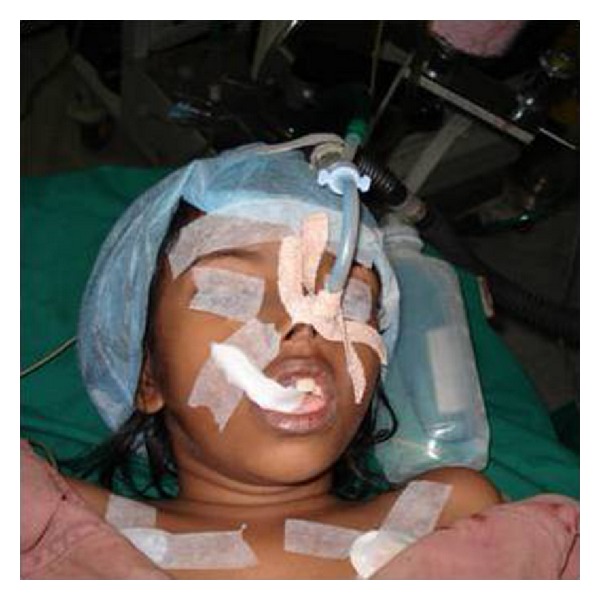
Photograph showing treatment procedures done under general anesthesia.

**Figure 7:**
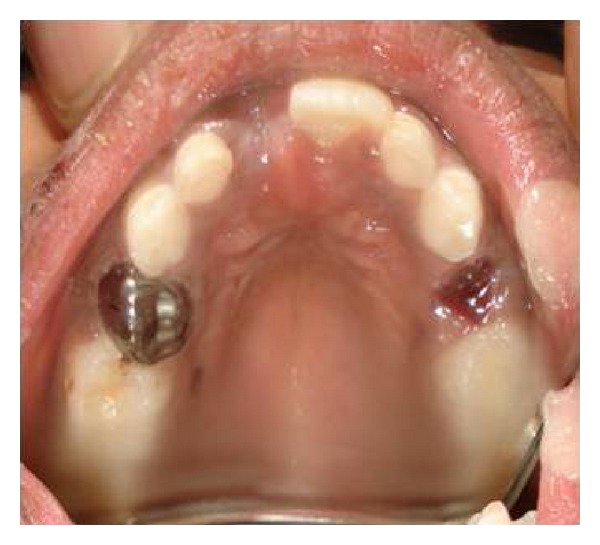
Intraoral postoperative photograph showing stainless steel crown in 54.

**Figure 8:**
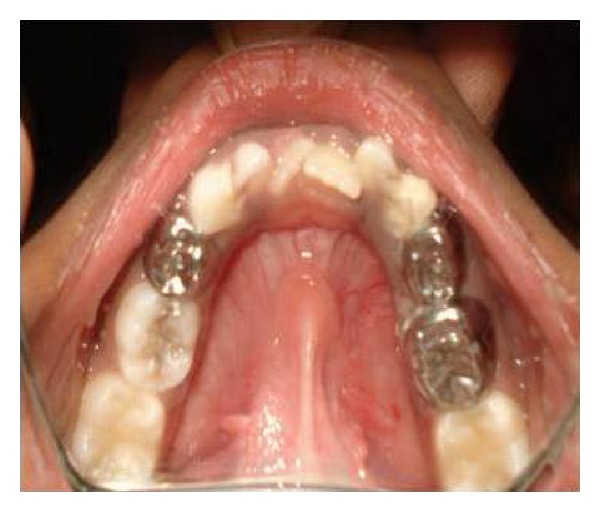
Intraoral postoperative photograph showing stainless steel crown in 74, 84, and 85 and ketac molar restoration in 83.

**Figure 9:**
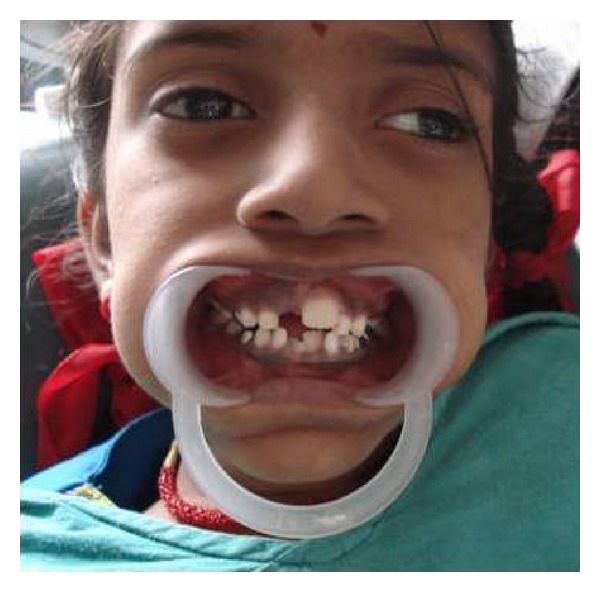
A one-month review photograph.

General examination revealed elongated fingers and toes (arachnodactyly) (Figures 1 and 2). On intraoral examination, and there was presence of high arch palate and dental caries in 52, 54, 62, 64, 73, 74, 82, 83, and 84. Class 1 malocclusion with crowding in lower anteriors was present. An OPG was taken (Figure 3), which showed dental caries involving the pulp in 54, 74, 83, 84, and 85. Resorption of roots was seen in 64. Pretreatment photographs were taken (Figures 4 and 5). Pulp therapy was done in 54, 73, 74, 75, and 84. The root canal obturation was done with metapex and access cavity was sealed with type IX GIC. Stainless steel crown was placed in 54, 74, 75, and 84 with type I GIC. Caries excavation was done using round bur in 52, 62, 72, and 83 and was restored with type IX GIC. Extraction of 64 was done under local anesthesia. Photograph was taken showing the treatment procedures under general anesthesia (Figure 6). Postoperative photographs were taken (Figures 7 and 8). The patient was reviewed after treatment for one day and she was discharged from the hospital. A one week review showed intact restorations and good retention of stainless steel crown. A one-month review of the child showed intact restorations and no signs of secondary caries (Figure 9).

## 3. Discussion

Childhood is a period of intensive physical and intellectual demands, self-assessment, and judgment by peers. Each of these processes can be influenced by the diagnosis of the Marfan syndrome. Parents and physicians should be sensitive to the cosmetic issues inherent in the Marfan syndrome and should anticipate or initiate discussions regarding potential solutions such as contact lenses or pectus repair. Finally, it may be useful to identify a role model or age-matched peer with the Marfan syndrome to discuss frustrations and opportunities with an affected child [8]. 

The eye evaluations should be performed every year. It is essential to identify and correct high refractive error or amblyopia in childhood in order to preserve and maximize visual function. Individuals with the Marfan syndrome are at increased risk for glaucoma, cataract formation, and retinal detachment, even in the absence of ectopia lentis. Progression of skeletal abnormalities, especially scoliosis and anterior chest irregularity, can be dramatic during periods of rapid growth, such as puberty. Evaluation and followup by an orthopedist is indicated in these cases. Children with the Marfan syndrome require frequent assessment of the status of the aortic root, with a maximal interval between echocardiograms of one year [9].

In children with Marfan's syndrome, a preventive management using battery operated brushes, adapted manual tooth brushes, fluoride products, and antibacterial mouth washes and toothpastes should be performed [10].

This paper describes one among the very rare syndromes affecting the children and adults. The dental findings may aid in diagnosis of this syndrome at earlier stage and it may aid in prompt multidisciplinary treatment.

## 4. Conclusion

In this case, the patient exhibits the orofacial features of Marfan syndrome such as crowded dentition and high arched palate. This is the rare paper of treating the dental problems in a child with Marfan syndrome. The dental problems of this child were successfully treated under general anesthesia and a one-month review showed intact stainless steel crowns restorations and no occurrence of secondary caries.
